# The combination of prostate imaging reporting and data system version 2 (PI-RADS v2) and periprostatic fat thickness on multi-parametric MRI to predict the presence of prostate cancer

**DOI:** 10.18632/oncotarget.17182

**Published:** 2017-04-18

**Authors:** Yudong Cao, Min Cao, Yuke Chen, Wei Yu, Yu Fan, Qing Liu, Ge Gao, Zheng Zhao, Xiaoying Wang, Jie Jin

**Affiliations:** ^1^ Department of Urology, Peking University First Hospital, Beijing, China; ^2^ Institute of Urology, Peking University, National Urological Cancer Center, Beijing, China; ^3^ Department of Radiology, Peking University First Hospital, Beijing, China

**Keywords:** periprostatic fat, prostate cancer, diagnosis, PI-RADS, nomogram

## Abstract

**Purpose:**

To evaluate the auxiliary effectiveness of periprostatic fat thickness (PPFT) on multi-parametric magnetic resonance imaging (mp-MRI) to Prostate Imaging Reporting and Data System version 2 (PI-RADS v2) in predicting the presence of prostate cancer (PCa) and high-grade prostate cancer (HGPCa, Gleason Score ≥ 7).

**Results:**

Overall, there were 371 patients (54.3%) with PCa and 292 patients (42.8%) with HGPCa. The mean value of PPFT was 4.04 mm. Multivariate analysis revealed that age, prostatic specific antigen (PSA), volume, PI-RADS score, and PPFT were independent predictors of PCa. All factors plus abnormal digital rectal exam were independent predictors of HGPCa. In addition, the PPFT was the independent predictor of PCa (Odds ratio [OR] 2.56, p = 0.004) and HGPCa (OR 2.70, p = 0.014) for subjects with PI-RADS grade 3. The present two nomograms based on multivariate analysis outperformed the single PI-RADS in aspects of predicting accuracy for PCa (area under the curve: 0.922 vs. 0.883, p = 0.029) and HGPCa (0.919 vs. 0.873, p = 0.007). Decision-curve analysis also indicated the favorable clinical utility of the present two nomograms.

**Materials and Methods:**

The clinical data of 683 patients who received transrectal ultrasound guided biopsy and prior mp-MRI were reviewed. PPFT was measured as the shortest perpendicular distance from the pubic symphysis to the prostate on MRI. Univariate and multivariate analyses were performed to determine the independent predictors of PCa and HGPCa. We also constructed two nomograms for predicting PCa and HGPCa based on the logistic regression.

**Conclusion:**

The PPFT on mp-MRI is an independent predictor of PCa and HGPCa, notably for patients with PI-RADS grade 3. The nomograms incorporated predictors of PPFT and PI-RADS demonstrated good predictive performance.

## INTRODUCTION

Prostate cancer (PCa) is the second most commonly diagnosed cancer among males worldwide [1]. Increasing age, ethnic background and heredity are well-established risk factors of PCa [2]. In addition, multiple epidemiological studies in recent years have suggested that obesity is associated with increased risk and death from numerous cancer types, including PCa [3–5].

The causal link between obesity and prostate carcinogenesis is fully expounded. Visceral fat is thought to play a prominent role in the tumor microenvironment as a metabolically active endocrine organ. Periprostatic fat (PPF), which surrounds the prostate, could produce several hormones and cytokines involved in autocrine, paracrine and endocrine signaling, such as vascular endothelial growth factor, tumor necrosis factor-α, interleukin-6, leptin and adiponectin [6–9]. Thus far, studies of PPF have yielded interesting findings. Several clinical studies show that periprostatic fat thickness (PPFT) is correlated with disease aggressiveness in patients diagnosed with PCa [10–13]. Moreover, Bhindi et al. discovered that PPFT could be a risk factor for the detection of prostate cancer and high-grade prostate cancer (HGPCa) among patients undergoing prostate biopsy procedures, which, to our knowledge, is the sole study in patients without prior diagnosis of PCa [14].

The measurement of PPF using various imaging tests may have contributed to the discrepant results. However, magnetic resonance imaging (MRI) is better able to characterize periprostatic adipose tissue in the retropubic area than transrectal ultrasonography (TRUS) and computed tomography (CT) [12–14]. In addition, pre-biopsy multi-parametric magnetic resonance imaging (mp-MRI) has exhibited great promise for the detection and characterization of prostate cancer [15–17]. The Prostate Imaging Reporting and Data System (PI-RADS), established by European Society of Urogenital Radiology (ESUR) in 2012 and updated to version 2 in 2014, was created to standardize the interpretation and systematic reporting of prostate MR imaging on a five-point scale [18–19]. Several literatures demonstrated that PI-RADS version 2 has shown substantial clinical utility in classifying the risk groups and improving the yield of the target biopsy [15–17, 20–22]. Hence, we wonder whether PPFT could be used to improve the diagnostic capacity of mp-MRI as an auxiliary geometric parameter combined with PI-RADS version 2 among the biopsy cohort.

Therefore, the present study used mp-MRI as a more accurate and feasible approach to measure PPFT and investigated whether it is a predictor of PCa and high-grade PCa. The study further estimated the auxiliary effectiveness of PPFT in diagnosis combined with PI-RADS version 2 score on mp-MRI. To our knowledge, this is the first study to discover the correlation between PPFT and PCa on MRI among a prostate biopsy cohort.

## RESULTS

### Patients’ demographics and baseline characteristics

**Figure 1:**
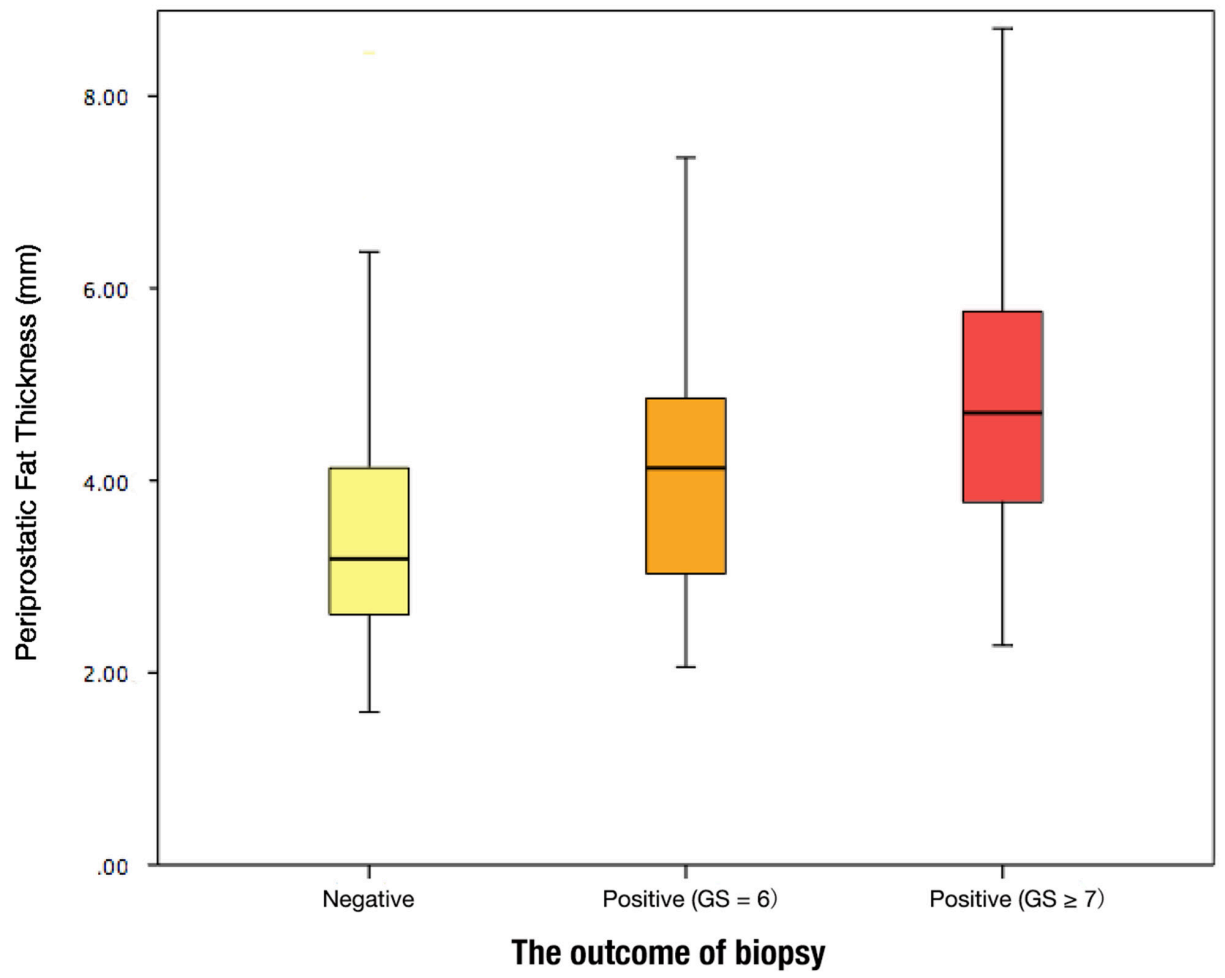
Periprostatic fat thickness (PPFT) distribution by the outcome of biopsy

The descriptive statistics of the study cohort were present in Table 1. Overall, 371 patients (54.3%) had prostate cancer and 292 patients (42.8%) had high-grade prostate cancer. The mean age and BMI were 64.96 years and 24.08 kg/m^2^, respectively. The mean periprostatic fat thickness and subcutaneous fat thickness measured on MRI were 4.04 mm (standard deviation 1.45 mm) and 24.85 mm (standard deviation 8.37 mm), respectively. The difference in the PPFT for the biopsy-negative cohort (mean 3.52 mm; standard deviation 1.26 mm; range 1.59 – 9.57 mm) vs. the PCa of the Gleason score = 6 (mean 4.09 mm; standard deviation 1.31 mm; range 2.06 – 7.61 mm) cohort and the PCa of the Gleason score ≥ 7 (mean 4.59 mm; standard deviation 1.48 mm; range 2.01 – 10.15 mm) cohort was highly statistically significant (p < 0.001, Figure 1). However, BMI was significantly correlated with subcutaneous fat (r = 0.577, p < 0.001). There was no significant relationship between BMI and periprostatic fat (r = -0.039, p = 0.314).

**Table 1:** Risk factors for presence of PCa and HGPCa based on univariate and multivariate analyses

	Total patients	Patients with prostate cancer				Patients with high-grade prostate cancer			
		Univariate analysis		Multivariate analysis		Univariate analysis		Multivariate analysis	
Variable	Value	Value	P*	Odds Ratios (95% CI)	P*	Value	P*	Odds Ratios (95% CI)	P*
Total, n (%)	683 (100)	371 (54.3)				292 (42.8)			
Mean (SD)									
Age, years	64.96 (8.67)	69.83 (8.18)	<0.001*	1.058 (1.027 - 1.090)	0.001*	70.04 (8.36)	<0.001*	1.029 (1.001 - 1.059)	0.048*
BMI, kg/m^2^	24.08 (2.77)	23.87 (3.04)	0.33	-	-	23.91 (3.12)	0.685	-	-
%fPSA***	0.171 (0.010)	0.12 (0.05-0.28)	<0.001*	-	-	0.11 (0.05-0.28)	<0.001*	-	-
PI-RADS score	3.56 (1.292)	4.40 (0.890)	<0.001*	3.200 (2.577 - 3.974)	<0.001*	4.58 (0.692)	<0.001*	3.080 (2.448 - 3.875)	<0.001*
SCFT, mm	24.85 (8.37)	24.83 (8.50)	0.945	-	-	25.10 (9.03)	0.692	-	-
PPFT, mm	4.04 (1.45)	4.48 (1.46)	<0.001*	1.549 (1.303 - 1.842)	<0.001*	4.59 (1.48)	<0.001*	1.467 (1.242 - 1.731)	<0.001*
Median (IQR)									
PSA level**, ng/ml	11.57 (7.54 - 20.84)	15.14 (9.25 - 36.02)	<0.001*	2.090 (1.535 - 2.845)	<0.001*	19.47 (10.54 - 44.95)	<0.001*	2.035 (1.530 - 2.705)	<0.001*
TPV**, ml	55.5 (39.0 - 80.0)	47.10 (35.1 - 67.0)	<0.001*	0.387 (0.237 - 0.632)	<0.001*	46.00 (35.2 - 66.9)	<0.001*	0.517 (0.322 - 0.831)	0.006*
Suspicious DRE, n	187	140	<0.001*	1.037 (0.571 - 1.884)	0.905	131	<0.001*	2.163 (1.269 - 3.687)	0.005*

* Statistically significant.

** PSA level and TPV were log transformed to approximate a normal distribution in binary logistic analysis.

*** %fPSA was not included in the multivariate analysis due to collinearity with PSA level.

PCa= prostate cancer; HGPCa= high-grade prostate cancer; BMI= body mass index; PSA= prostate specific antigen; %fPSA = percentage of free PSA; PI-RADS= prostate imaging reporting and data system; TPV= total prostate volume; SPFT= subcutaneous fat thickness; PPFT= periprostatic fat thickness; DRE= digital rectal exam.

### Univariate and multivariate analyses of risk factors for presence of PCa and HGPCa

Based on univariate analysis, PPFT, age, prostatic specific antigen (PSA), the percentage of free-PSA (%fPSA), total prostate volume (TPV), PI-RADS score and suspicious digital rectal exam (DRE) demonstrated statistical significance between biopsy-negative patients and PCa patients as well as biopsy-negative patients and HGPCa patients based on univariate analysis. Using a multivariate logistic regression model to estimate effect of variables, age, PSA, TPV, PI-RADS score, and PPFT were significant predictors of PCa. All of the above factors plus suspicious DRE were also significant predictors for high-grade prostate cancer (Table 1). For each millimeter increase in PPFT, there was a 55% (OR 1.55, 95% CI 1.03–1.84) and 46% (OR 1.46, 95% CI 1.20–1.73) increase in the odds of detecting prostate cancer and high-grade prostate cancer, respectively.

Likewise, univariate and multivariate analysis of the prediction of PCa and HGPCa were performed in the only ‘indeterminate’ PI-RADS grade 3 subgroup (78 patients). %fPSA and PPFT demonstrated significance between biopsy-negative patients and PCa patients as well as HGPCa patients. Moreover, age, %fPSA and PPFT were independent predictors of PCa and HGPCa. For each millimeter increase in PPFT, there was a 156% (OR 2.56, 95% CI 1.35–4.83) and 170% (OR 2.70, 95% CI 1.27–5.77) increase in the odds of detecting prostate cancer and high-grade prostate cancer, respectively.

### Development and the comparison of nomograms predicting the presence of PCa and HGPCa

**Figure 2:**
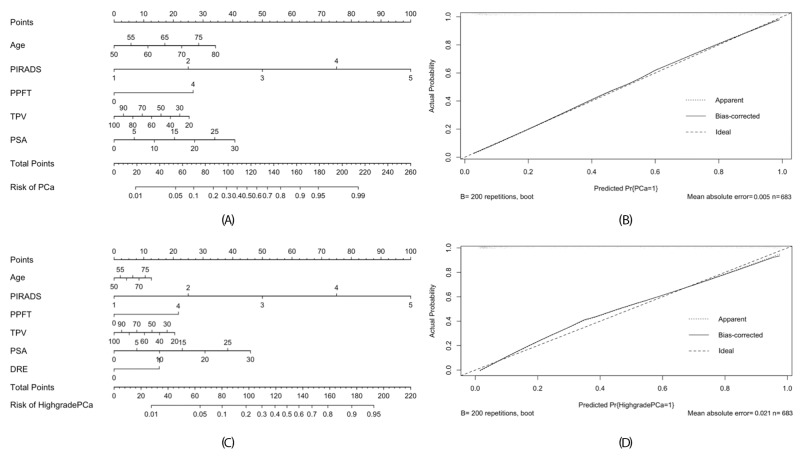
Nomogram **(A)** and calibration plot **(B)** for predicting detecting PCa of model 1, and nomogram **(C)** and calibration plot **(D)** for predicting detecting HGPCa of model 2. For easily application, the PPFT was defined as a categorical variable at the threshold of 4 mm using the Youden criterion.

Based on multivariate analysis, we developed corresponding nomograms and calibration plots for the prediction of PCa and HGPCa (Figure 2). The calibration plots for both models were not far from ideal. The AUCs for model 1 predicting prostate cancer was 0.922 (95% CI 0.901–0.943) and model 2 predicting high-grade prostate cancer was 0.919 (95% CI 0.89–0.94). The accuracies were significantly higher than the AUCs of the PI-RADS score (0.922 vs. 0.883, p= 0.029 and 0.919 vs. 0.873, p = 0.007, respectively).

### Decision curve analysis

**Figure 3:**
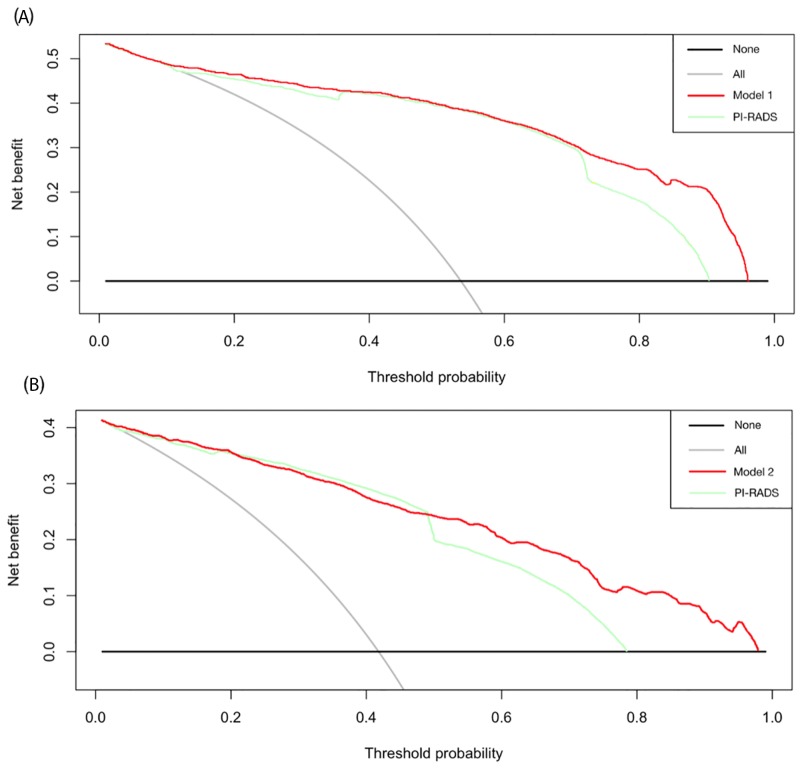
Decision curves of **(A)** the prostate cancer predictability of the model 1 and PI-RADS score, and **(B)** high-grade disease predictability of the model 2 and PI-RADS score. Decision analysis demonstrated a high net benefit for the model 1 and the model 2 compared to PI-RADS alone (p=0.029, p=0.007, respectively).

In Figure 3, the results of the decision curve analysis (DCA) of PCa and HGPCa predictability for the two models are presented. Using decision-curve analysis, Model 1 had a superior net benefit in the range of 72% to 96%, while Model 2 had a superior net benefit in the range of 48% to 97%, which suggested the favorable clinical utility of the two models relative to PI-RADS with these ranges.

## DISCUSSION

The prostate is a capsule-like organ surrounded by periprostatic fat tissue, which is regarded as a metabolically active organ. According to its potential role in the tumor microenvironment, the link between periprostatic fat and tumorigenesis as well as the tumor progression of prostate cancer has attracted attention. Most studies demonstrated a correlation of PPF and the aggressiveness of PCa based on the post-operative histological confirmed cohort [10–13]. In the sole study of patients without prior prostate cancer diagnosis, Bhindi et al. measured PPFT on transrectal ultrasonography and identified it as a risk factor for detecting prostate cancer and high-grade prostate cancer among patients undergoing prostate biopsy procedures. However, the further role of PPFT in clinical practice and decision-making remains unknown.

In the present study, we demonstrated that PPFT was the independent predictor of prostate cancer and high-grade prostate cancer on MRI. Increasing PPF thickness was found to be a risk factor for detecting prostate cancer as well as high-grade disease upon biopsy. For each millimeter increase in PPF thickness, there was a 55% and 46% increase in odds of detecting prostate cancer and high-grade prostate cancer, respectively. The odds of risk are higher than data reported by Bhindi et al., reflecting a stronger correlation between PPFT and malignant disease [14]. In ROC analysis, the AUCs of PPFT were comparable to other classical clinical parameters, including age, PSA, %fPSA, TPV and DRE; however, they were significantly less than PI-RADS score.

Studies have shown the clinical utility of PI-RADS in localizing prostate cancer, classifying the risk groups, and improving the yield of target biopsy since it was introduced by the European Society of Urogenital Radiology [22]. Hamoen et al. reported that PI-RADS showed a sensitivity of 0.78 and specificity of 0.79 for PCa detection, presenting good diagnostic accuracy [23]. However, a major limitation of the clinical application of the PI-RADS system is the tendency to score a lesion “3,” making it indeterminate of clinical choice instead of a binary decision. In the present study, PPFT was confirmed as the independent risk factor for PCa and HGPCa. A millimeter increase in PPFT was associated with 156% increased odds of detecting prostate cancer and 170% increased odds of high-grade prostate cancer. This strong correlation supports the use of PPFT in the further stratification of risk in the PI-RADS grade 3 subgroup.

To investigate the availability and utilization of PPFT together with other significant parameters, including PI-RADS score, to predict a positive prostate biopsy in clinical practice, we developed nomograms to provide a more accurate assessment of the risk of detecting prostate cancer and high-grade prostate cancer based on a Chinese population. Published nomograms for predicting biopsy results were generally constructed by predictors including age, PSA, %fPSA, prostate volume (PV) and DRE. Fang et al. incorporated PI-RADS score on pre-biopsy MRI into nomograms showed a good diagnostic performance of the accuracies of detecting prostate cancer (87.5%) and high-grade prostate cancer (87.2%), suggesting that the pre-biopsy MRI could increase predictive accuracy. [24] In our models that included PPFT and PI-RADS as both parameters performed on mp-MRI, the accuracy of detecting prostate cancer and high-grade prostate cancer was 92.2% and 91.9%, respectively, which were significantly superior to the single PI-RADS score as well as any other variable. The predictive accuracies exhibited good performance compared to previous studies. [25–28] Our nomograms provide numerical estimate calculators combining the PPFT and PI-RADS score as well as other variables to inform clinical decision-making. However, further external validation is required to confirm the utility of our models.

Limited by subjectivity and the non-determinacy of the real-time procedure, ultrasonography is a less appropriate method to measure and quantify fat tissue. Accelerated acquisition, quantitative reconstruction and a physiologically based threshold make MRI a more clinically feasible and appropriate method to distinguish and quantify fat tissue. [29] Moreover, a pre-biopsy mp-MRI is becoming more recommended and utilized in order to help select candidates for biopsy as well as proceed MRI-target biopsy, which makes it possible to measure PPFT before biopsy. [15–17, 30–31] Thus, the feasibility and practicality of PPFT measured by a pre-biopsy mp-MRI make it a promising novel clinical parameter in the prediction of prostate biopsy outcome.

In the present study, BMI and subcutaneous fat thickness measured on MRI were not correlated with the detection of PCa and HGPCa in the overall cohort. Moreover, BMI showed a significant relationship with subcutaneous fat but not periprostatic fat thickness. Our findings are generally consistent with previously published literature [12]. We suggest that periprostatic fat, regarded as metabolically active visceral fat, is a distinct parameter instead of a surrogate marker for general obesity. Previous studies showed conflicting results in the link between PPF and tumorigenesis of PCa [7–9, 32]. Further studies about the role of PPF in prostate carcinogenesis will be required to elucidate their association.

Several limitations of our study exist. First, it was a retrospective analysis. Second, the measurement of PPF thickness was only performed in one plane on MRI. A more accurate approach, such as volumetric quantification, may be required to establish a standardized method. Third, the nomograms we developed have not been validated by external databases since the PPFT is not a universal parameter in clinical practice. However, the results of our study demonstrated that PPFT is a promising predictor of prostate biopsy detection. A further step would be to evaluate the utility of our models.

## MATERIALS AND METHODS

### Subject selection

Between January 2013 and December 2015, a total of 764 patients who underwent pre-biopsy prostate MRI and TRUS-guided prostate biopsy performed within 3 months at our institution (Peking University First Hospital) were initially collected. Of these patients, 81 were excluded according to the following criteria: (a) history of previous prostate biopsy (n = 45), (b) history of hormonal therapy (n = 16) before biopsy, (c) poor image quality on mp-MRI (n = 11), and (d) lack of detailed clinical information (n = 9). Therefore, 683 patients were enrolled for evaluation. This study was approved by the Ethics Committee of Peking University First Hospital.

### Clinical and pathological data

The following clinical information was evaluated from medical records: age; height and weight; body mass index (BMI); results of digital rectal exam (DRE); prostatic specific antigen(PSA) levels measured before DRE and TRUS, including the percentage of free-PSA (%fPSA); total prostate volume (TPV) determined by TRUS; data from TRUS-guided prostate systematic needle biopsies; and prostate mp-MRI findings.

All biopsy specimens were evaluated by a dedicated genitourinary pathologist to determine the presence of PCa and the Gleason score in positive cases. The outcome variable of the study was the presence of prostate cancer upon biopsy. Patients were classified as either having no prostate cancer or low- (Gleason score = 6) and high- (Gleason score ≥ 7) grade prostate cancer.

### MRI protocol

MRI images were acquired by one of the following three 3.0T scanners (Intera Archieva, Philips Medical System; Discovery MR750, GE Medical Systems; Signa HD, GE Medical Systems) 4-6 weeks before transrectal ultrasonoguided biopsy. These protocols included dual-echo T1-weighted imaging in the sagittal planes, fast-spin-echo T2- weighted imaging in the axial planes, diffusion-weighted imaging in the axial plane (*b* values: 0, 800, and 1000 sec/mm^2^), and dynamic contrast-enhanced imaging with the main MR imaging acquisition parameters described in Supplementary Table 1. The ADC map was generated from the DW imaging, with *b* values of 0 and 800 sec/mm^2^. The patients were asked to take some type of laxative one day before the examinations. No endorectal coil was used.

### Image analysis

Two radiologists (A[GG] and B[XW]) who were experienced with PI-RADS version 2 and had 4 and 15 years of experience in prostate MRI reviewed the images separately at a picture archiving and communication system (PACS). Both radiologists were not informed of the patients’ clinical data. They scored the images following the standards of PI-RADS version 2 with T2-weighted imaging, diffusion-weighted imaging and dynamic contrast enhanced imaging [19].

**Figure 4:**
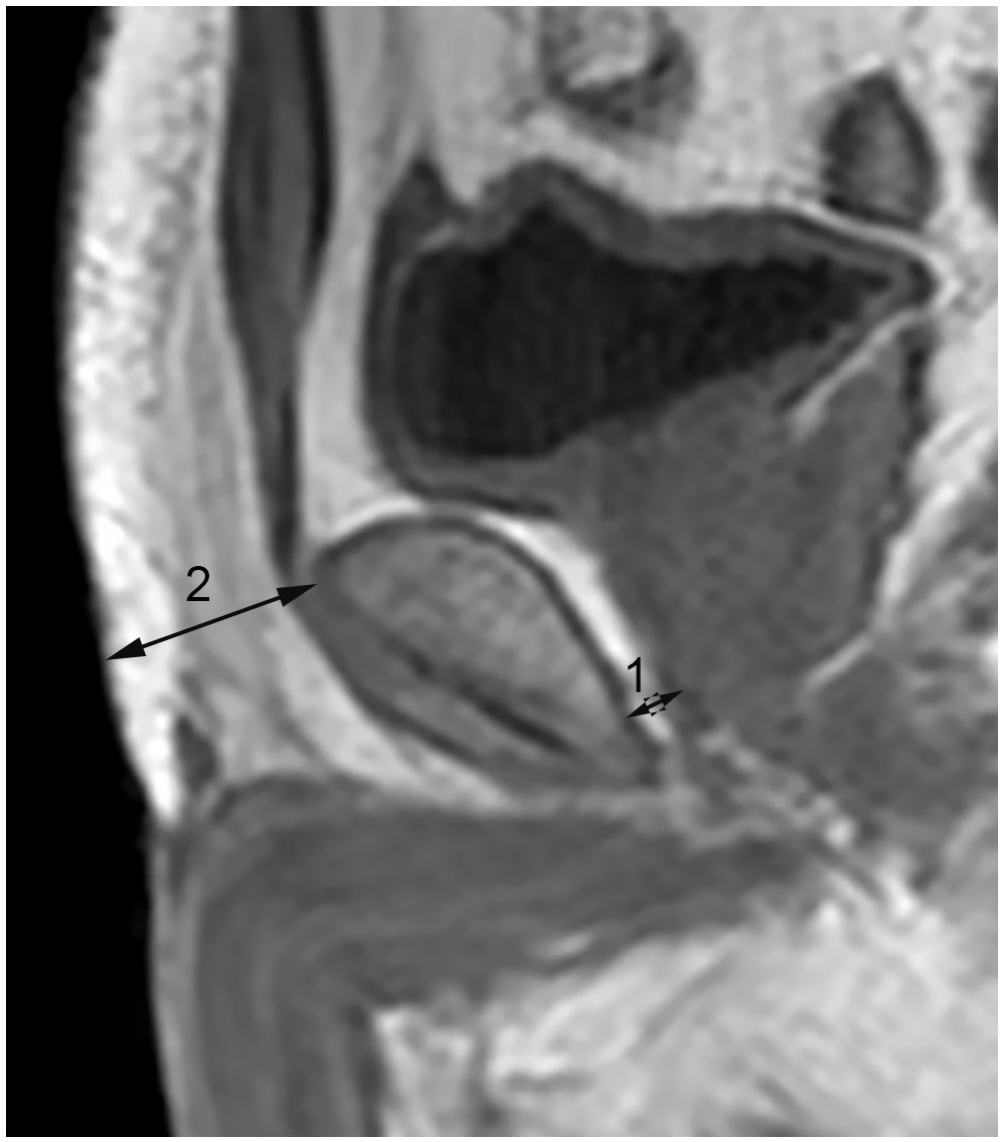
Subcutaneous and periprostatic fat thickness measurement on midsagittal dual-echo T1 weighted imaging Line 1: Periprostatic fat thickness. Line 2: Subcutaneous fat thickness.

Two radiology residents (C[MC] and D[QL]) with no experience with PI-RADS version 2 and no knowledge of the clinical information of the patients measured the subcutaneous and periprostatic fat thickness on dual-echo T1 weighted imaging. Subcutaneous fat thickness was defined as the shortest distance from the top of the pubic subcutaneous to the surface of the abdominal wall[12]. Periprostatic fat thickness was defined as the shortest distance between the posterior margin of the pubic symphysis and the superior margin of the prostate at the midsagittal plane, as shown in Figure 4 [12, 14]. The present measurement approach we used had favorable repeatability and stability, which could minimize the measurement errors from different planes and avoid interference of morphological changes of periprostatic hollow organs, including the bladder and rectum.

### Statistical analysis

Categorical variables were analyzed using the chi-square test or Fisher's exact test, while continuous variables were analyzed using the independent T test, analysis of variance and the Mann–Whitney U test. The Pearson correlation coefficient (*r*) was used to test for correlation between BMI and subcutaneous or periprostatic fat thickness. Binary logistic regression was performed to calculate the odds ratios for the predictive factors of prostate cancer and high-grade prostate cancer. To approximate a normal distribution for improving the model, PSA values and TPV were log transformed in logistic analysis.

For better application in individual risk evaluation, two predictive models were constructed to predict the presence of PCa (Model 1) and HGPCa (Model 2) based on multivariable binary logistic analysis. Model 1 was constructed by clinical factors that exhibited a statistical association including age, PSA, PI-RADS, PPFT, and TPV, whereas Model 2 added DRE results. Discrimination was measured using the area under the curve (AUC) of the receiver operating characteristic (ROC) curve. Calibration plots were performed to examine the performance characteristics of the risk calculators. The comparison of AUCs and decision curve analysis was also performed.

The generation of the nomograms, calibration plots and DCA curves was performed with the statistical software package R version 3.1.3 (R foundation for Statistical Computing, Vienna, Austria), and other statistical tests were performed with SPSS 21.0 (IBM Corp, USA). *p <* 0.05 was considered to indicate statistical significance.

## CONCLUSIONS

Periprostatic fat thickness measured on mp-MRI was an independent predictor of detecting prostate cancer and high-grade prostate cancer upon biopsy, notably in the PI-RADS grade 3 subgroup. The nomograms that incorporated PPFT and PI-RADS v2 score demonstrated good performance in predicting an individual risk of prostate cancer or high-grade disease upon biopsy.

## SUPPLEMENTARY MATERIALS TABLE



## Abbreviations

PCa: prostate cancer
HGPCa: high-grade prostate cancer
BMI: body mass index
PSA: prostate specific antigen
%fPSA: percentage of free PSA
PI-RADS: prostate imaging reporting and data system
TPV: total prostate volume
SPFT: subcutaneous fat thickness
PPFT: periprostatic fat thickness
DRE: digital rectal exam.
