# Supplementary material for: The combination of prostate imaging reporting and data system version 2 (PI-RADS v2) and periprostatic fat thickness on multi-parametric MRI to predict the presence of prostate cancer
Source: Oncotarget. 2017 Apr 18;8(27):44040–9. doi: 10.18632/oncotarget.17182 (PMC5546460; doi:10.18632/oncotarget.17182)
Supplement: Supplementary file 1 [file oncotarget-08-44040-s001.pdf]

The combination of prostate imaging reporting and data system version 2 (PI-RADS v2) and periprostatic fat thickness on multi-parametric MRI to predict the presence of prostate cancer

SUPPLEMENTARY MATERIALS

SUPPLEMENTARY TABLE

Supplementary Table 1: MRI Imaging Protocols

	Intera archieva				Discovery MR750, Signa HD			
**Parameters**	**T1WI**	**T2WI**	**DWI**	**DCE**	**T1WI**	**T2WI**	**DWI**	**DCE**
Repetiton time (msec)	195	2900	4000	3.3	195	4600	4000	3.3
Echo time (msec)	2.4	90	70	1.6	2.4	100	70	1.6
Flip angle (degree)	75	90,180	90	15	75	90×80	90	15
Matrix	320×224	320×280	184×184	256×256	320×224	320×240	184×184	256×256
Field of view (mm)	360×360	260×260	260×260	260×260	360×360	260×260	260×260	260×260
No. of signal acquired	1	4	4	0.75	1	2	4	0.75
Section thickness (mm)	6	4	4	2	6	4	4	2
Spacing (mm)	0.5	-	-	-	0.5	-	-	-
b value (sec/mm2)		-	0, 800, 1000	-			0, 800	-
